# Supplementary material for: MetaQTL: a package of new computational methods for the meta-analysis of QTL mapping experiments
Source: BMC Bioinformatics. 2007 Feb 8;8:49. doi: 10.1186/1471-2105-8-49 (PMC1808479; doi:10.1186/1471-2105-8-49)
Supplement: Additional File 7 — MetaQTL Package : jar file and tutorial. This Zip archive contains both the MetaQTL JAR file and the files of the tutorial. [file 1471-2105-8-49-S7.zip › org.inra.metaqtl/doc/org/metaqtl/factory/MetaQtlAnalysisFactory.html]

MetaQtlAnalysisFactory


|  |  |  |
| --- | --- | --- |
| **Overview** \| **Package** \| **Class** \| **Use** \| **Tree** \| **Deprecated** \| **Index** \| **Help** | |  |
| **PREV CLASS**   **NEXT CLASS** | **FRAMES**    **NO FRAMES**     **All Classes** |  |
| SUMMARY: NESTED \| FIELD \| CONSTR \| METHOD | DETAIL: FIELD \| CONSTR \| METHOD |  |


---


## org.metaqtl.factory Class MetaQtlAnalysisFactory

```
java.lang.Object
  org.metaqtl.factory.MetaQtlAnalysisFactory
```

---

``` public final class MetaQtlAnalysisFactory extends java.lang.Object ```

A factory to deal with the result of a QTL meta-analysis.

---

**Constructor Summary**

`MetaQtlAnalysisFactory()`


| **Method Summary** | |
| --- | --- |
| `static MetaQtlAnalysis` | `read(java.io.InputStream stream)` |
| `static MetaQtlAnalysis` | `read(java.io.Reader reader)` |
| `static void` | `write(MetaQtlAnalysis analysis, java.io.OutputStream stream)`             This methods writes out in the given `stream` the results of a QTL meta-analysis. |

**Methods inherited from class java.lang.Object**

`clone, equals, finalize, getClass, hashCode, notify, notifyAll, toString, wait, wait, wait`

**Constructor Detail**

### MetaQtlAnalysisFactory

```
public MetaQtlAnalysisFactory()
```


**Method Detail**

### read

```
public static MetaQtlAnalysis read(java.io.InputStream stream)
                            throws java.io.IOException
```

:   **Parameters:**: `stream` - **Returns:**: **Throws:**: `java.io.IOException`

---


### read

```
public static MetaQtlAnalysis read(java.io.Reader reader)
                            throws java.io.IOException
```

:   **Parameters:**: `reader` - **Returns:**: **Throws:**: `java.io.IOException`

---


### write

```
public static void write(MetaQtlAnalysis analysis,
                         java.io.OutputStream stream)
                  throws java.io.IOException
```

:   This methods writes out in the given `stream` the results
    of a QTL meta-analysis. The user is responsible for closing the stream.

    :   **Parameters:**: `analysis` - the meta analysis results: `stream` - an output stream. **Throws:**: `java.io.IOException`


---


|  |  |  |
| --- | --- | --- |
| **Overview** \| **Package** \| **Class** \| **Use** \| **Tree** \| **Deprecated** \| **Index** \| **Help** | |  |
| **PREV CLASS**   **NEXT CLASS** | **FRAMES**    **NO FRAMES**     **All Classes** |  |
| SUMMARY: NESTED \| FIELD \| CONSTR \| METHOD | DETAIL: FIELD \| CONSTR \| METHOD |  |


---
